# Marketing in the era of COVID-19

**DOI:** 10.1007/s43039-020-00016-3

**Published:** 2020-11-23

**Authors:** Janny C. Hoekstra, Peter S. H. Leeflang

**Affiliations:** 1 Department of Marketing, University of Groningen, Groningen, The Netherlands; 2 Emeritus Frank M. Bass Professor of Marketing, University of Groningen, Groningen, The Netherlands; 3 Aston Business School, Birmingham, UK; 4 Member of the Royal Academy of Arts and Sciences, Amsterdam, The Netherlands

**Keywords:** Downcycle, Marketing strategy, Marketing policy, Consumer behaviour

## Abstract

We discuss the effects of COVID-19 on consumer behaviour and elaborate on the consequences of this disruption for marketing strategies and marketing policies. The crisis shows similarities with changes in consumer behaviour and the way marketing is carried out during economic downturns. However, it also displays characteristics which differ from downcycles, such as shifts in consumption between categories and the accelerated shift from offline to online behaviour. This is forced by the re-evaluation of life priorities by final consumers.

## Introduction

**Fig. 1:**
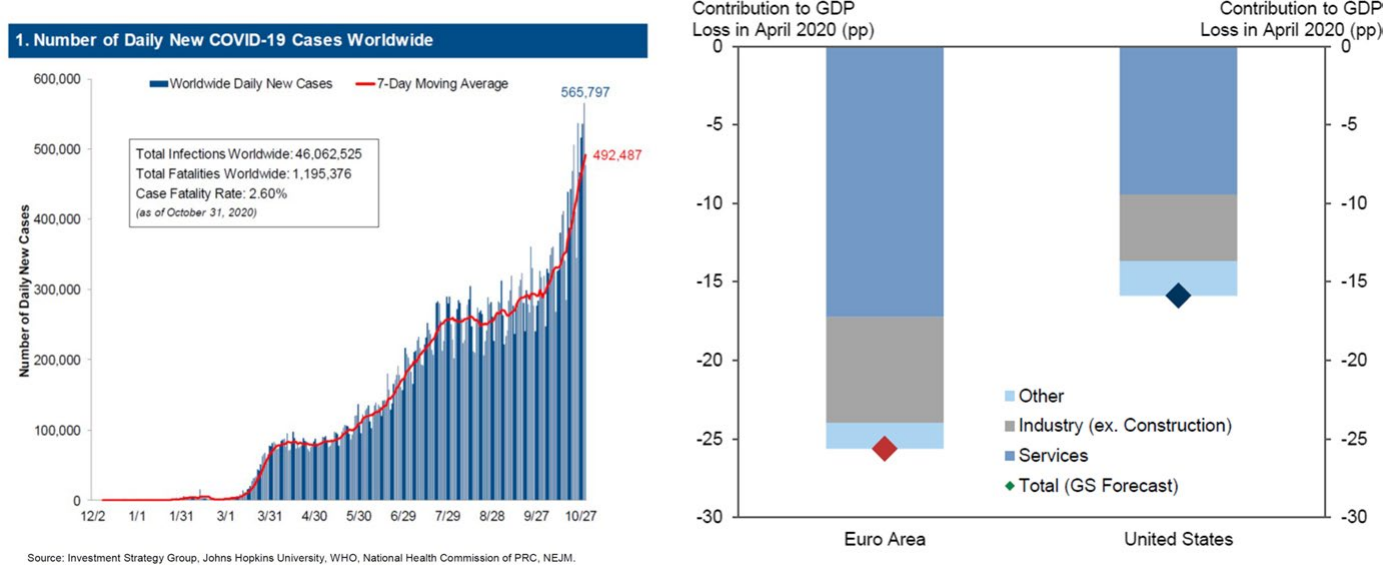
COVID-19 and GDP. *Source*: Goldman Sachs (2020)

The world as we know it is currently experiencing one of the greatest challenges since the Second World War. The COVID-19 crisis is affecting every aspect of our lives. We all feel compassion for those who are directly affected by the coronavirus. Society and the economy have largely been brought to a standstill, and almost every country is in the grip of a recession. Figure 1 illustrates how COVID-19 is affecting real GDP worldwide.

The left-hand side of Fig. 1 demonstrates that worldwide COVID-19 steadily increases over time. From the right-hand side of Fig. 1 we may also conclude that the worldwide downturn can be expected to have long-term effects. It is likely that the COVID-19 crisis will have substantial consequences for our way of living, working and shopping, and more specifically for consumer behaviour. This means it will affect almost all businesses. To be able to continue meeting consumers’ basic needs, and to maintain employment levels, companies must limit the damage as much as possible. One of the tools available to achieve this is marketing.

In this article on marketing and COVID-19, we discuss what previous research has revealed about how companies can adapt their marketing policy in times of crisis. We first discuss the effects of the unprecedented disruption caused by COVID-19 on immediate and long-term consumer behaviour. We subsequently elaborate on the consequences of this disruption for marketing strategies and marketing policies.

## The impact of COVID-19 on consumer behaviour

The COVID-19 crisis is affecting consumer behaviour and thus the way in which marketing can be used. The use of marketing during (and after) the COVID-19 crisis shows (and will continue to show) similarities with the way that marketing is carried out during economic downturns. Dekimpe and Deleersnyder (2018) have summarized the most relevant studies on the effectiveness of marketing efforts during downturns and upturns. However, this specific crisis, which will be followed by a recession (contraction), displays characteristics that differ from those associated with a recession. For example, as well as a fall in consumption due to lower consumer confidence, lower incomes, consumer defaults on loans and reduced financial means as a result of falling share prices, shifts in consumption are also occurring between product categories. In parallel to these economic developments, the imposition of social distancing is also affecting the drivers of consumer behaviour.

Consumers are being challenged to re-evaluate their life priorities, which may give rise to new values and spending criteria. In this respect, Euromonitor International (2020a, b) has observed a focus on family/community/self, health and digital solutions, and expects this to last into the long term. More specifically, Euromonitor has identified various megatrends, including:

1. Connected Consumers: both consumers and businesses are showing stronger emotional connections with reliable suppliers in their search for stability and value. In this respect, digitalization is more important than ever and shows how consumers, employers and employees may be able to keep operating in the future. Services such as Zoom and Google Meet are proving to be indispensable in many more situations than we were previously aware of. Moral and ethical values (referred to as ethical living) are also receiving greater attention. In relation to this, Euromonitor has mentioned the tendency of consumers to become more engaged with products and services, and to attach greater value to connections with reliable, often important and well-known brands such as IKEA, Knorr, Maggi and Disney. We also observe that consumers are looking for reliable information about COVID-19 and its consequences. This is, for example, reflected in greater attention being paid to non-commercial websites and TV broadcasting.
2. Healthy Living: a healthy lifestyle and healthy habits inside and outside the home are becoming more important, and a more holistic approach to wellness is being adopted.
3. Middle Class and Lower Class Retreat: as a result of COVID-19 and its economic consequences, we observe that the middle and lower economic classes are struggling to maintain their economic position and lifestyles. We observe that unemployment in the USA is increasing dramatically, and is affecting about 25% of all US citizens. In Africa, the situation is even worse. Middle class retreat can be observed in Europe in particular. This is leading to behaviours such as sharing products (Eckhardt et al. 2019), renting and borrowing.
4. Shopping Reinvented: social distancing is leading to a shift to online shopping among many consumers. This includes both groceries and durables. As a result, online stores are experiencing enormous increases in turnover. We also observe that consumers who were not previously familiar with online shopping are now becoming ambassadors for this way of shopping. The expectation is that at least a part of the shift to online shopping will be permanent. The COVID-19 crisis has also led to a move towards buying more locally produced food. For example, the Streetify e-commerce platform brings together buyers and local stores.
5. Shifting Market Frontiers: we observe that large cities reach saturation when social distance has to be maintained. The space offered by free zones (parks, avenues, shopping streets) and nature (such as woods and beaches) is insufficient. As a result, a shift to living in mid-size cities is expected. It is also believed that certain markets have reached their limits, both directly as a result of COVID-19-related measures (including the travel industry, particularly aviation and cruises, and the restaurant industry), and indirectly, as a result of the developments mentioned above (such as farming (both intensive livestock breeding and intensive agriculture), mining and fast food).

**Fig. 2:**
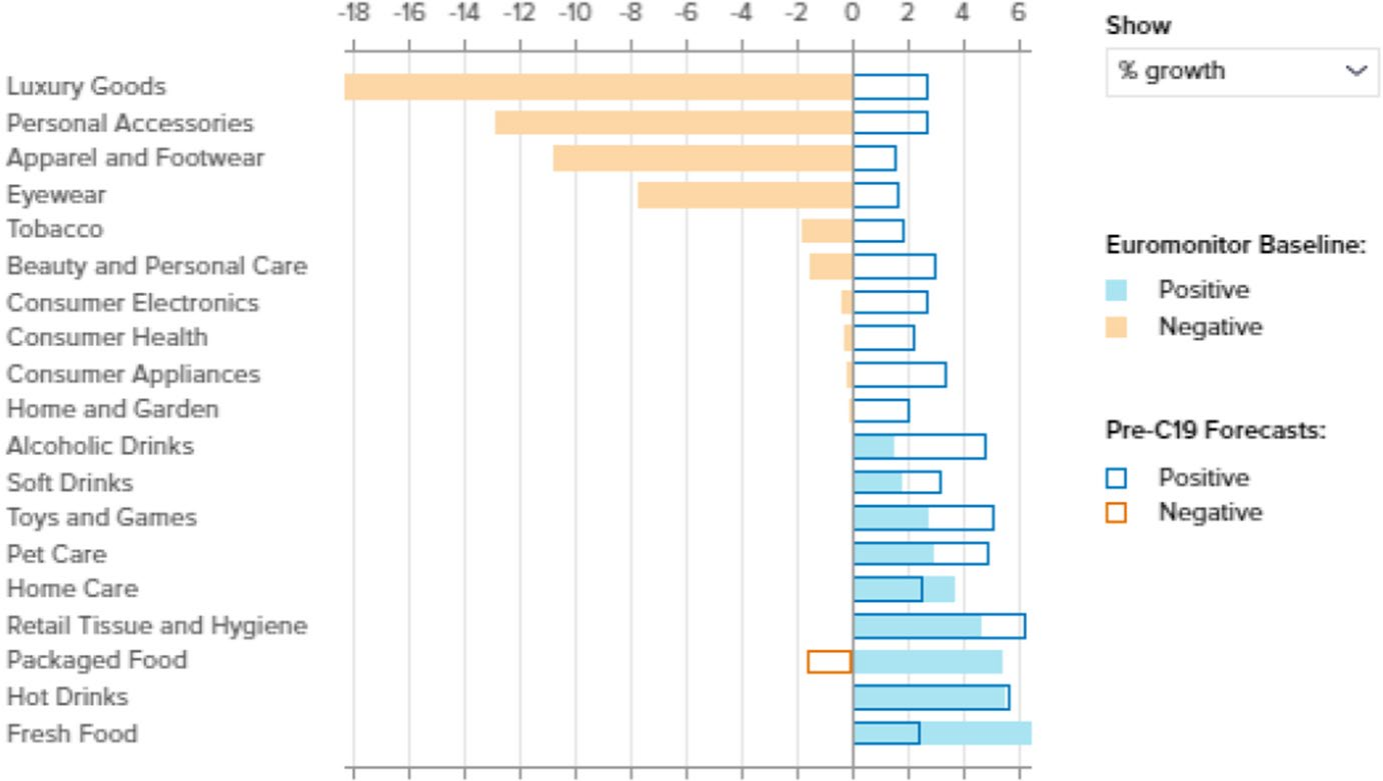
The impact of COVID-19 on retail sales. *Source*: Euromonitor International (2020b, p. 7)

We observe changes in consumer spending as a consequence of these trends. This is illustrated in Fig. 2.

Figure 2 illustrates that, with the exception of fresh food, packaged food and home care, COVID-19 is expected to negatively affect consumer markets. Other data sources show increases in infotainment (Netflix, for example, attracted about 16 million new subscribers between April and June. Games, puzzles, etc. are also increasingly popular: the board game Trekking the World generated revenues of about $100 K in 1 week), hygiene products and wellness, digital products, gardening materials, do-it-yourself products (+ 25%), streaming services, furniture (+ 8%), and consumer electronics (16%).^1^ All this reflects the feeling that the home is a healthy bubble.

Many of these and other products are bought online. As an example: in the Netherlands, sales by online stores in the non-food sector rose by 60–70% in April 2020 (compared to normal growth of 16–18%), according to the GfK market research agency. In May 2020, online sales were 50% higher than in May 2019.^2^

Substantial decreases in consumer sales have occurred in areas such as cars (sales are 40–50% down on the same period last year), shoes (down 45%), clothes (down 60%, even strong brands such as G-star and Nike are facing much lower sales and the Belgium/Netherlands/Scandinavia-based FNG chain is close to bankruptcy), visits to restaurants and bars (hence also alcoholic consumption, which has resulted, for example, in a major loss for Heineken in the 2nd quarter of 2020) and travel and outdoor recreation (events, museums).

How can marketers respond to the disruption caused by the pandemic and the contraction in the economy?

## The impact of COVID-19 on strategic decision-making

The Spring 2020 issue of AMA’s Marketing News gives many examples of how leading marketers are working during the pandemic. Many managers employ a short-term view and are having difficulties ensuring that their new, short-term actions still fall in line with their longer-term strategic plans. Companies are also urged to calibrate and redefine their Purposes, Products, Channels and target Customers at this time.

### Purpose

First of all, we observe that companies are adapting their goals and are launching initiatives designed to contribute to tackling COVID-19. Such activities are referred to as ‘purpose marketing’ or ‘cause-related marketing’ and demonstrate corporate social responsibility (CSR). We observe many calls for purpose marketing to help elderly and vulnerable people in society and to support and encourage those in need of emotional support. Although research shows that announcing cause-related activities may negatively influence shareholder value (Woodroof et al. 2019), research also shows that such activities result in more positive consumer attitudes and larger purchase intentions (e.g., Trimble and Rifon 2006). Companies that engage in COVID-19 related activities either have the resources to do this and/or are able to benefit from the shifts in demand occurring during the pandemic. For example, Coca-Cola has donated 120 million dollars to fighting COVID-19 and temporarily halted its commercial activities. Nivea (Beiersdorf) has donated 50 million and Facebook 100 million. Toyota Netherlands is using its dealer network to collect and distribute medical and other supplies. Another example is a supermarket chain that is donating ten eurocents to the Red Cross for every carton of milk, porridge and custard sold. In a meta-analysis, Fan et al. (2020) find that the effect of cause-related marketing is larger when it is done by a familiar brand of a utilitarian product, when a relatively large amount of money is donated and when the cause is less familiar. It is also important that such activities connect with the authenticity of the brands that initiate them. If this is the case, these companies will not only be doing a good deed: these activities will also strengthen the consumers’ brand attachment and word-of-mouth (Morhart et al. 2015).

### Product

Many companies have developed great out-of-the box ideas that redefine their product portfolios. Examples include:

- DSM, a manufacturer of plastics and nutritional products, now produces face masks, as does Auping, a manufacturer of mattresses.
- A dry cleaner that uses disinfection cabinets to disinfect clothes has established a new company in partnership with other market players to manufacture disinfection cabinets. These are subsequently sold to hospitals.
- Hooghoudt, a distiller of alcoholic beverages such as jenever and lemonade syrups, now also produces hand sanitizer.
- In Italy, artificial snow cannons have been re-purposed as aerosol machines for disinfection.
- Many restaurants have developed take-away services. An upscale Seattle-based restaurant has transformed into three pop-up restaurants: a drive-through burger restaurant, a bagel shop and a family meal delivery service.
- The delivery service Deliveroo, which primarily handles restaurant orders, now also delivers products from Marks and Spencer.

Research has shown that it is wise to invest in R&D and innovations in times of contraction (Srinivasan et al. 2011; Steenkamp and Fang 2013), not only in terms of products but also new (potentially complementary) services and processes, such as the aforementioned home delivery service. The receptiveness of consumers to new products/services is greater during periods of contraction than during periods of expansion. In addition, in such times, many R&D departments have the opportunity to work on new products without excessive time constraints and with greater creativity. This particularly applies to companies that rely less on R&D capacity to resolve production problems. Communication about investments and the results of R&D also contributes to greater appreciation of the company by investors (see for example Edeling and Fischer 2016).

### Channel

During the COVID-19 crisis, marketing is faced with a major challenge: how do we get the products to buyers? Distribution is limited and many retail chains (IKEA, clothing chains) have even been forced to (temporarily) close their doors. Companies with their own online channel are at an advantage compared to stores that only operate offline. Research shows that companies that apply a multichannel strategy in which they combine offline and online channels, perform better in terms of share of wallet (Melis et al. 2016) and in terms of revenues (Pauwels and Neslin 2015). Such companies reacted better during the pandemic because they were already prepared to offer their products and services online where others were not, and were therefore more responsive to changes in the customer journey (Lemon and Verhoef 2016). At the same time, the increased demand for their products also necessitates creative solutions. For example, a popular department store in the Netherlands (HEMA, where online sales have trebled) is using around twenty stores as distribution centres to avoid long waiting times at the central distribution centre. The Rituals cosmetic chain has chosen a similar strategy (300% increase in online sales). Deliveries of products ordered by customers in the local area are also being made by bicycle. Some stores are also offering digital sales advice, for example via WhatsApp or FaceTime. Concerts and theatre performances are also being streamed live.

Companies without their own online channel can offer products online by making use of existing platforms. In the United States, this is occurring on a massive scale via platforms such as Podia, Sellfly and Sendowl. Services such as seminars, education and consultancy services are also being distributed via these platforms.

The COVID-19 crisis has also made it painfully clear how dependent we are on foreign markets, on both the demand and supply side. For example, the supply of semi-finished and finished products from China has ceased temporarily and foreign sales of cut flowers have also stopped. Innovations can focus on a reduction of these dependencies. On the supply side, 3D printing (of plastic and other synthetic materials, wood, copper, resin etc.) offers new possibilities. Semi-finished and finished products no longer need to be shipped but can be produced (printed) locally. On the demand side, we observe many online and offline ‘buy local’ initiatives, which allow manufacturers to generate sales where this would otherwise be impossible. For example, local retailers can make (temporary or permanent) use of platforms such as the aforementioned Streetify to offer their wares, either for pickup or home delivery. In general, we observe a tendency to shorten supply chains.

### Target customers

Although research has shown that communication in times of contraction favours national brands (see below under ‘Marketing policies’), advertising, especially in mass media, has nevertheless declined dramatically. Globally, we observe a decline in advertising expenditure of about 10%,^3^ with television advertising expenditure decreasing by 25%. Communication is directed less at acquiring new customers; instead, most companies are devoting greater attention to their existing customers. There are, however, also companies that increase their investments in advertising. Examples are Proctor and Gamble and Unilever, companies that are less impacted by COVID-19 given the wide arrange of products for daily use. The same can be observed when we consider the more intense communication of grocery retailers and retailers of do-it-yourself products, furniture, cosmetics, etcetera. It is suggested to use combinations of classical and social media, given that these two types of media offer opportunities for synergy when they are used in combination (De Vries et al. 2017). Another option that is quite often used is sending e-mails. As one of the leaders in the AMA’s leaders’ survey has pointed out: ‘Proactive emails with personalized ideas and strategy recommendations have been appreciated and have generated new opportunities with current clients’ (Steimer 2020, p. 51). One of the conclusions of the survey is that one-to-one personalized communication is most effective. However, communication is complex, because—regardless of the communication channel used—there is now a lot more noise and everyone seems to be saying the same thing.

However, the COVID-19 crisis also offers opportunities for customer acquisition. The increase in the use of the online channel allows companies to collect data about new customers. This data can be used to profile these customers (e.g., to compare them with similar customers based on their buying behaviour), possibly leading to the identification of new target groups. The next step is to target these new customers with suitable or even customized offers.

## The impact of COVID-19 on marketing policies

In the previous section we discussed the most relevant directions for evaluating and changing marketing strategies in the era of COVID-19. In this section we discuss some suggestions for modifying marketing policies in the fields of assortment, private labels, price and price promotion, and communication.

### Assortment

Many companies are using the COVID-19 crisis to critically evaluate the size (breadth and depth) of their range to increase profitability. Sloot et al. (2006) have shown that offering a substantial number of products does not contribute to profitability. The authors assessed the short-term and long-term effects of a 25% reduction in items on category sales. They found that a major range reduction can lead to substantial short-term category sales losses but has only a weakly negative effect on long-term sales. The effect on customer profitability and lifetime value is unknown. These may decrease if certain products are no longer available and customers decide to do some or all of their shopping elsewhere. Hence, it may be very useful to reconsider the range of products that are offered now. COVID-19 indicates the need for critically evaluating the supply of products in the assortment. This is also caused by possible changes in how we reconsider our use of products and services in the post-COVID-19 time period. For example, do we really need all those short-distance flight connections or can these flights be skipped and replaced by train connections? And are people still wearing business costumes and suits now they are so much accustomed to wearing casual clothes?

### Private labels

The share taken by private labels increases during downturns at the expense of national brands and shrinks when the economy is flourishing (Lamey et al. 2007). Even after a downturn, the share taken by branded products is generally lower than it was before the downturn. As such, it is necessary to invest in branded products. Given the Euromonitor megatrends discussed earlier in this paper, this is especially relevant for national and international brands that are authentic, transparent and safe. The sustainability of the brand also plays a major role, as the COVID-19 crisis coincides with the ongoing discussions on climate change and its consequences for our planet and its inhabitants.

### Price and price promotions

Price must be used with great care as a marketing instrument during this crisis. The price sensitivity of consumers increases during a contraction (Van Heerde et al. 2013). The extent to which price sensitivity increases is dependent on various factors. How unique is the product? How important is the product to consumers? And does the company focus on a mass market or a ‘niche’? Brands that focus on a mass market and that are less unique are more sensitive to price than more unique brands that focus on niches. Now more than ever, it is all about creating products that offer the consumer ‘value for money’. Consumers will be displeased if they have to pay more without a concurrent increase in product and/or service quality (Hunneman 2020).

Temporary discount offers can be used to respond to increased price sensitivity. At the same time, price promotions can lead to a price war and a lower reference price (the price that consumers expect to pay for a product). Suppliers that want to reduce the risks of such effects may make use of types of promotion other than price, for example by offering gifts and (possibly additional) services. Volume discounts are out of the question as the current crisis has led to undesirable hoarding of products.

### Communication

In times of crisis, companies tend to reduce spending on communication/advertising. Deleersnyder et al. (2009) have shown that advertising expenditure is considerably more sensitive to business fluctuations than the economy as a whole. Moreover, the growth in private label sales is greater in countries characterized by more cyclical advertising spending, implying significant losses for brand manufacturers. Research has shown that the sensitivity to advertising can be greater in a period of contraction than in a period of expansion (Steenkamp and Fang 2013). Van Heerde et al. (2013) confirmed that this is the case for foodstuffs, but not for drinks. As such, generalizations about this are not possible. However, companies that maintain communication spending during a contraction can win a greater ‘share of voice’ in the market if competitors communicate less (or not at all) during the same period.

Supporting brands with communication during a period of contraction shows that:

1. in such cases, consumers are less likely to switch from national brands to private labels;
2. following a period of contraction, these brands can regain the market position they had before the contraction/crisis more rapidly and at a lower cost (Van Heerde et al. 2007).

The content of communication must be adapted to the current situation. When products are (temporarily) unavailable, promotional advertising does not make sense. This specific crisis period also demands the use of different media: less outdoor advertising, more online, and possibly more television and radio advertising. AMA chapter leaders believe that e-mails are more effective than messages on social media. Recent problems with Facebook, Twitter and YouTube also support these sentiments. Some managers use highly targeted unique direct mailings to reach their customers (Steimer 2020, p. 50).

Personal approaches focused on ‘how can we help you’ give insight into the challenges faced by customers and offer opportunities to help them. However, ‘we are here for you’ messages are only effective if they are authentic and are followed up. This not only affects marketing communication. It requires businesses to be fully aligned with the customer-centric paradigm (see for example Hoekstra et al. 1999), which is not (yet) the case for many businesses. The time is ripe for companies to respond to the immediate, basic needs of their customers. For example, various suppliers have adapted their communication to these times of social distancing and ‘caring for one another’. The careful use of humour can also help to increase the retention of the advertising message.

Companies that feel the need to reduce their advertising costs can make greater use of ‘contact advertising’ (blogs, vlogs, articles, press releases, newsletters, updating the website, writing books). Communications in which a brand demonstrates concern for victims of the coronavirus, or responds to the regulations imposed to tackle the crisis, will only have a positive effect on the brand image if they are authentic and connect with the brand identity.

It is possible to reallocate resources by having sales staff carry out other duties, for example assisting with the development of new product ideas and making contributions to content marketing. Now is the time to improve the connection between sales and marketing. As one of the marketing leaders said: ‘Sales processes and lead generation have had to change a lot with social distancing, so we’ve actively pursued ways to help our clients overcome these challenges with an increased digital presence and ways to hold sales presentations virtually’ (Steimer 2020, p. 53).

## Conclusion and future research

The current crisis can also teach us a lot about our recent history. Many companies have a limited vision of what efficiency means. They primarily focus on short-term cost reductions at the micro level, and devote little or no attention to the time, energy and (ethical background of) production factors used in their operational processes. This limited approach has been taken to an excessive degree at some companies, resulting in large-scale outsourcing instead of in-house production, low stock levels, a high degree of dependence on foreign manufacturers (e.g., in China) and low prices (such as for groceries).

In the future, we will have to pay more for our daily groceries and for many services, including healthcare. If this leads to a reduction in demand for products that threaten the future of our planet (transport/travel, for example), this would in itself be a benefit. It may be hoped that the current developments will lead to creative innovations that will contribute to a more sustainable and ethical economy and society in the post-COVID-19 era.

This era should also stimulate researchers to investigate changes in the relevant stages in consumer behaviour, both with regard to (the pre-)buying (process) and with regard to use and the disposal of the products and services. We hypothesise that COVID-19 has influenced the steps in the customer journey (Lemon and Verhoef 2016) for many consumers and many products, but we are not aware of any study in this area. We also need more insights and knowledge with regard to changes in consumer behaviour that are persistent and that are transient. Persistence modelling offers an excellent tool to distribute demand effects over these two components (e.g., see Osinga et al. 2010). This era also affects the way in which we use and dispose of our products, and how we share products (Eckhardt et al. 2019).

To conclude, this era offers many opportunities for marketers to demonstrate their value for companies which either are hurt by COVID-19 or are doing well by facilitating how we deal with the pandemic. The same holds for marketing scientists who study transient and permanent effects of COVID-19 on consumer behaviour and how marketing strategies and marketing policies can be adapted by organizations.

## Footnotes

1 Statistics Netherlands, 2020.

2 Statistics Netherlands, 2020.

3 https://www.zenithmedia.com/coronavirus-crisis-accelerates-shift-to-digital-advertising/.
